# Supplementary figures and images for: Single-cell RNA profiling of Plasmodium vivax-infected hepatocytes reveals parasite- and host- specific transcriptomic signatures and therapeutic targets
Source: Front Cell Infect Microbiol. 2022 Aug 25;12:986314. doi: 10.3389/fcimb.2022.986314 (PMC9453201; doi:10.3389/fcimb.2022.986314)

Figure S1

Replicate 2  
Day 5 post infection

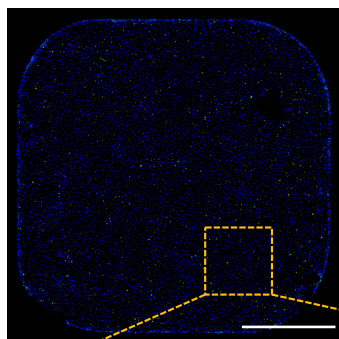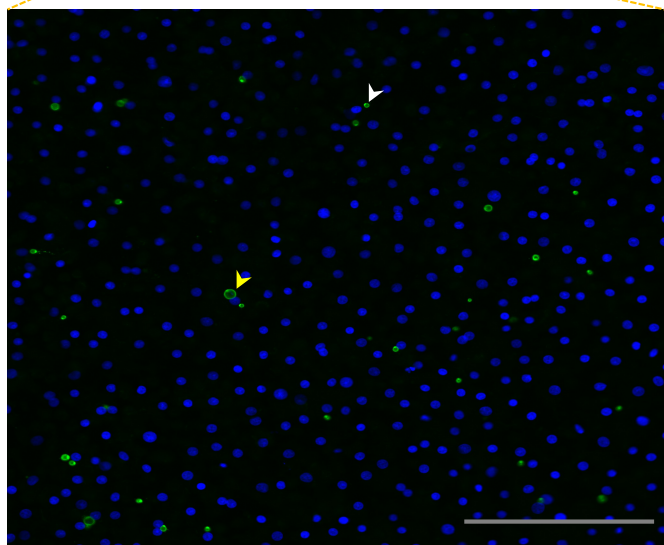

Day 9 post infection

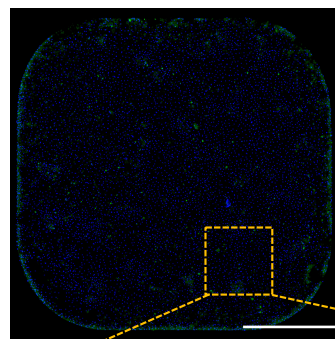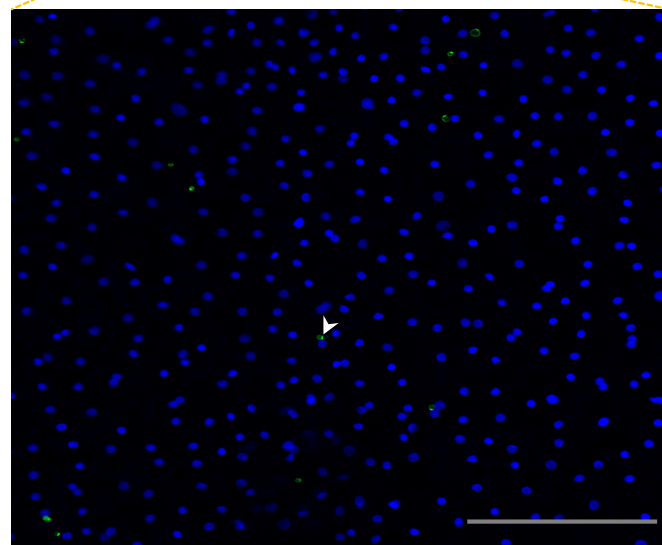

Supplement: Supplementary Figure 1 — Assessment of P. vivax liver stages using high-content imaging. Representative high content images of primary hepatocytes infected with P. vivax on day 5 (left) and day 9 (right) post-infection. Images were obtained from one well from a 384-well plate with a 4x objective. Inset: one field of view (orange box) from the same well captured with a 20x objective. Cells were stained with DAPI (blue) and PvUIS4 (green). White arrow: liver form assigned as a hypnozoite, yellow arrow: liver form assigned as a schizont. White bar represents 1mm, grey bar represents 200µm. Images are representative of the second biological replicate used in the study. [file Image_1.pdf]

Figure S2

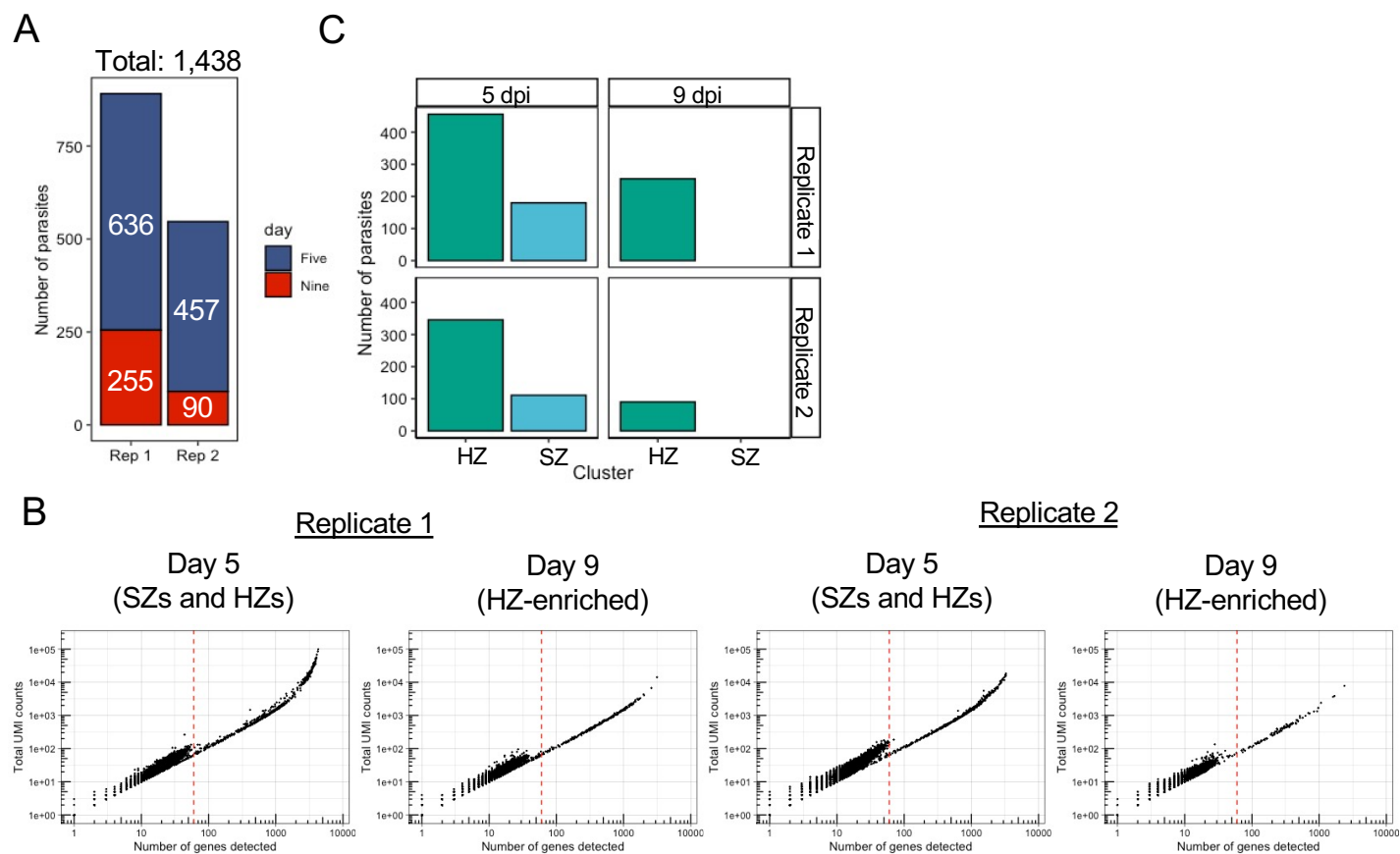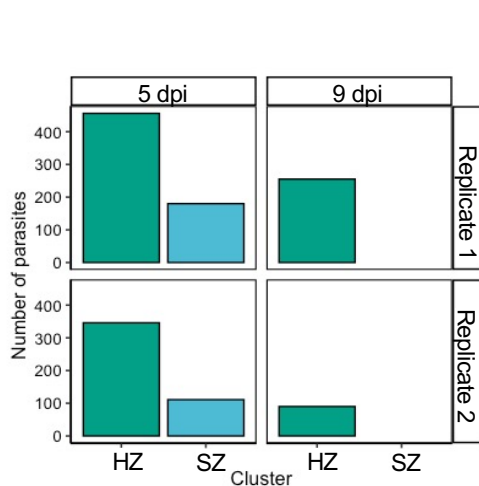

Supplement: Supplementary Figure 2 — Metrics associated with P. vivax liver stage scRNA-seq data analyses. (A) Number of P. vivax liver stage transcriptomes assessed post-cell and gene filtering. (B) Scatter plots displaying the number of genes detected versus total number of UMIs for each parasite transcriptome assessed. Red dashed vertical line (60) represents the cut-off used to filter viable from problematic (dead/dying/poorly captured) cells. (C) Number of P. vivax liver stage parasites assigned to cluster 1 (hypnozoites) and cluster 2 (schizonts). SZ: schizont; HZ: hypnozoite; dpi: day post-infection. [file Image_2.pdf]

Figure S3

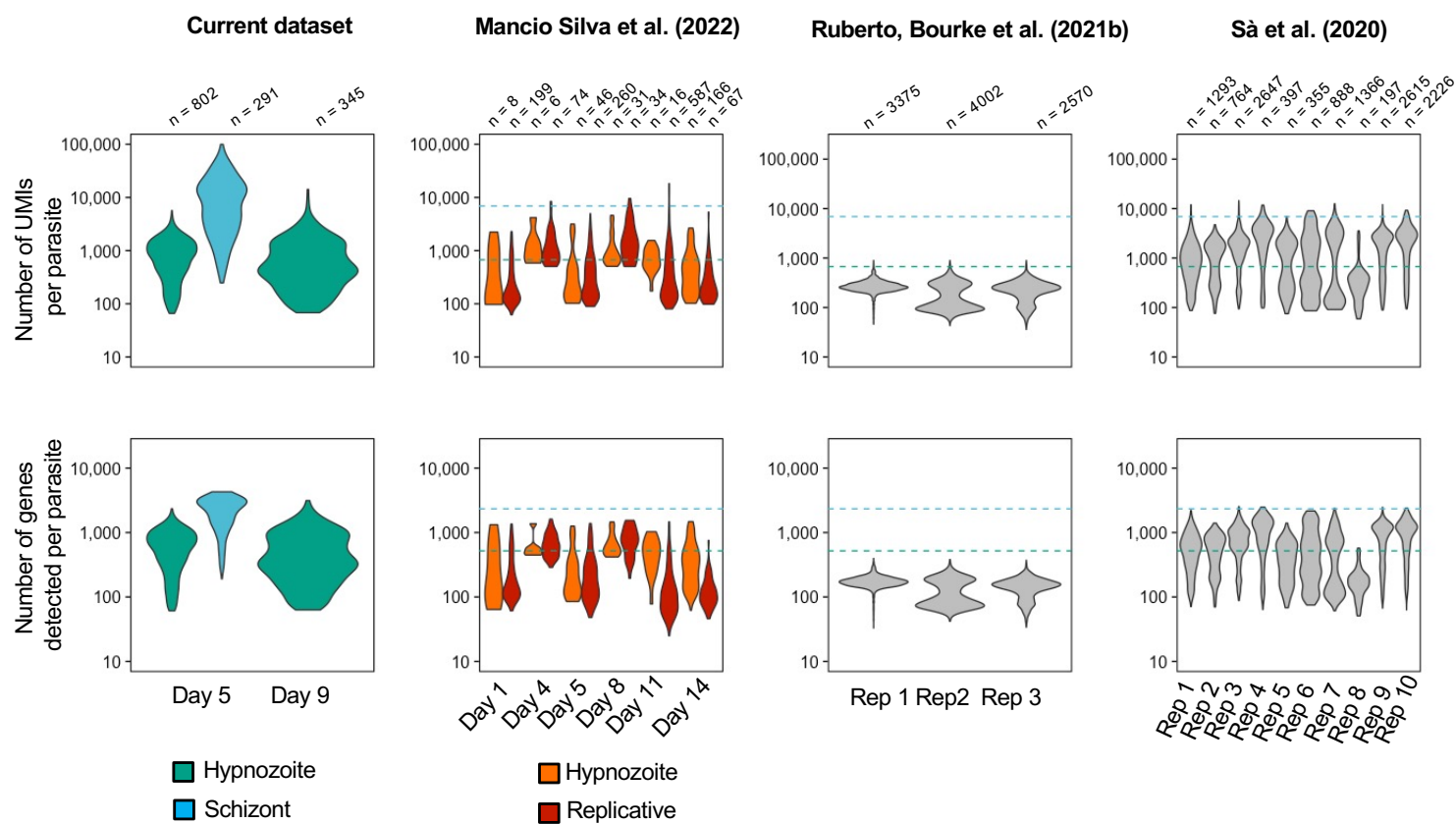

Supplement: Supplementary Figure 3 — Comparison of per cell metrics with other P. vivax scRNA-seq data. Violin plots showing the distribution of genes detected in the current dataset, and other single-cell gene expression assessments of P. vivax liver stages (Mancio-Silva et al., 2022), sporozoites (Ruberto et al., 2022), and blood-stage parasites (Sà et al., 2020). Replicates in sporozoite and blood-stage datasets represent unique 10x Genomics’ scRNA-seq library preparations. Blood-stage scRNA-seq metrics were obtained after realignment of sequencing data to an updated P. vivax transcriptome including UTRs as performed previously (Ruberto et al., 2022). N = number of single-cell transcriptomes assessed for each sample. Light blue dashed horizontal line: median number genes detected or UMIs in schizonts obtained in the current dataset; teal dashed horizontal line: median number genes detected or UMIs in hypnozoites obtained in the current dataset. Day denotes day post-infection. [file Image_3.pdf]

Figure S4

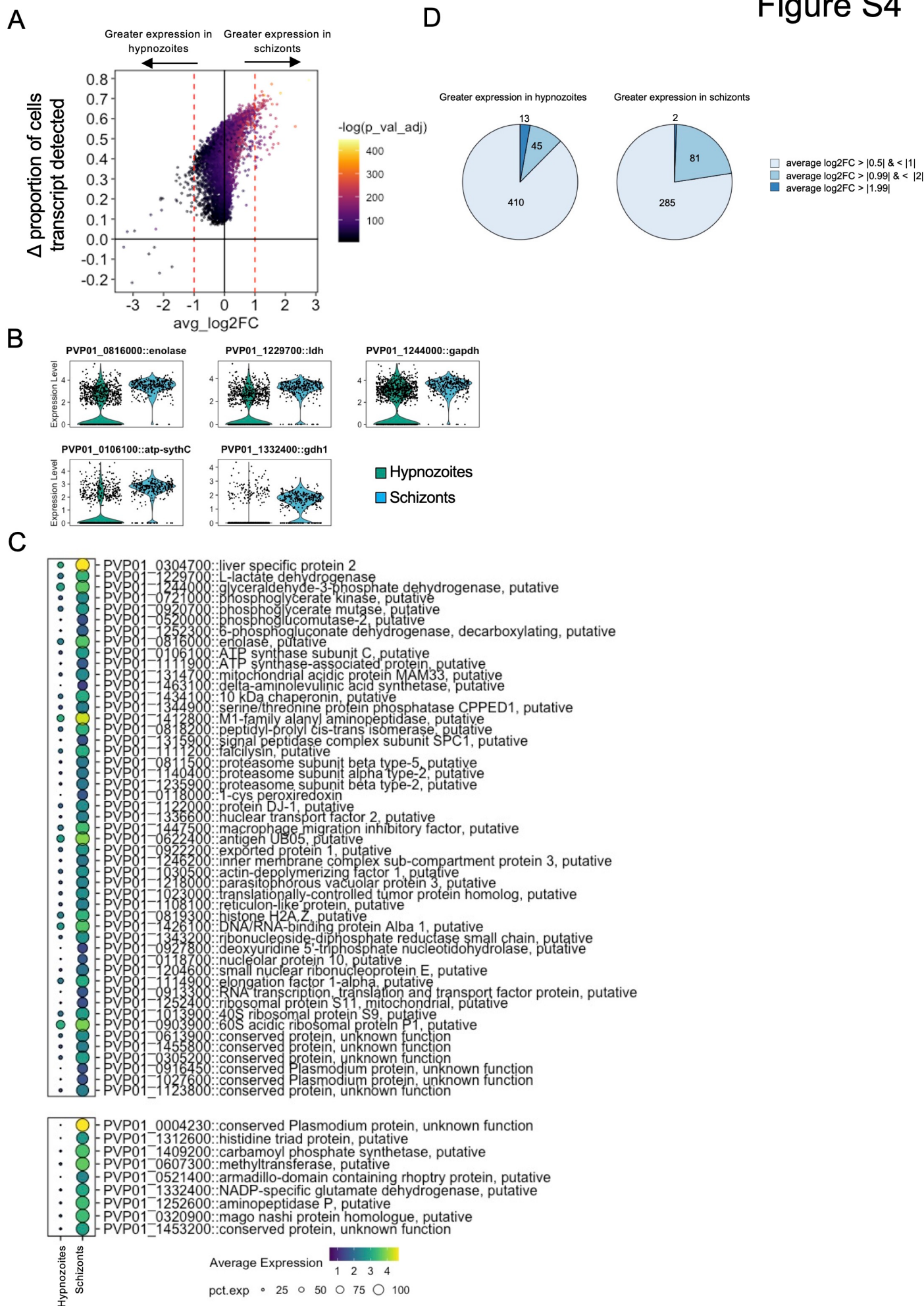

Supplement: Supplementary Figure 4 — P. vivax schizonts and hypnozoites have distinct transcriptomic signatures. (A) Scatterplot showing average log2 fold change versus the difference in the proportion of cells the transcript is detected in clusters encoding for hypnozoites and schizonts. Negative values: greater detection in hypnozoites; positive values: greater detection in schizonts. (B) Violin plots displaying the expression of genes encoding for TCA- and glycolysis-related proteins. (C) Dot plots showing transcripts with decreased expression in hypnozoites relative to schizonts. The size of the dot corresponds to the percentage of cells expressing the gene, colored by average expression. Differentially expressed transcripts were identified using Seurat’s FindMarkers function. Wilcoxon rank-sum test, Bonferroni adjusted p values < 0.01. Scale: normalized expression; pct. exp: percent of cells expressing the gene. ldh: L-lactate dehydrogenase; gapdh: glyceraldehyde 3-phosphate dehydrogenase; atp-synthC: ATP synthase subunit C; gdh1: NADP-specific glutamate dehydrogenase 1. [file Image_4.pdf]

A

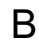C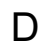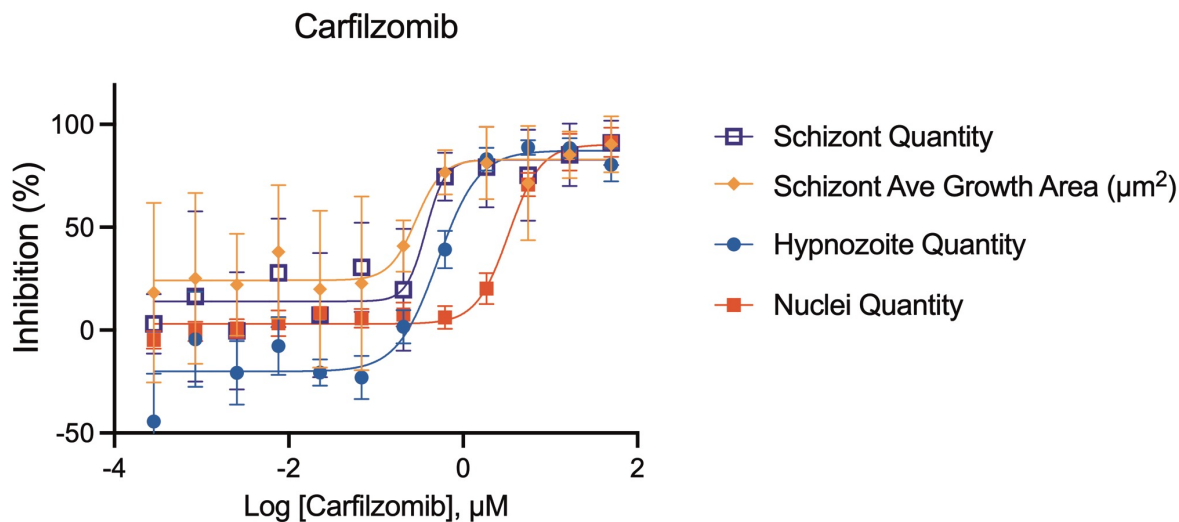

Supplement: Supplementary Figure 5 — Carfilzomib inhibits P. vivax schizonts and hypnozoites. (A) Violin plots displaying the distribution of expression of select genes encoding for proteasome subunits in schizonts and hypnozoites. (B) Table of proteasome-associated transcripts differentially expressed between schizonts and hypnozoites. Positive average log2FC: higher expression in schizonts. (C) Structure of carfilzomib. (D) Dose-response curve of carfilzomib-induced inhibition of P. vivax schizont quantity per well (purple, IC50 373 nM), schizont net growth area per well (yellow, IC50 288nM), hypnozoite quantity per well (blue, IC50 511nM), and hepatocyte nuclei quantity (orange, IC50 3.32 µM). Data shown are pooled from three independent experiments, each containing duplicate wells at each concentration. Bars represent ± SEM. [file Image_5.pdf]

Figure S6

A

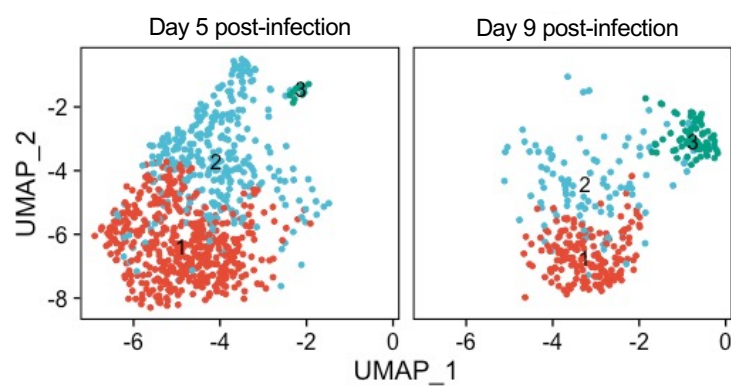

B

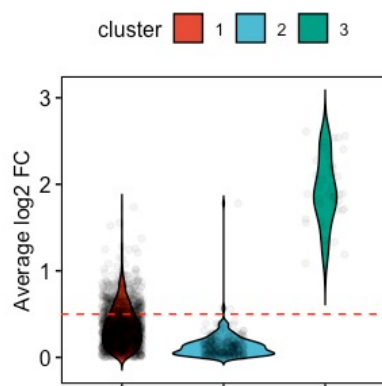

C

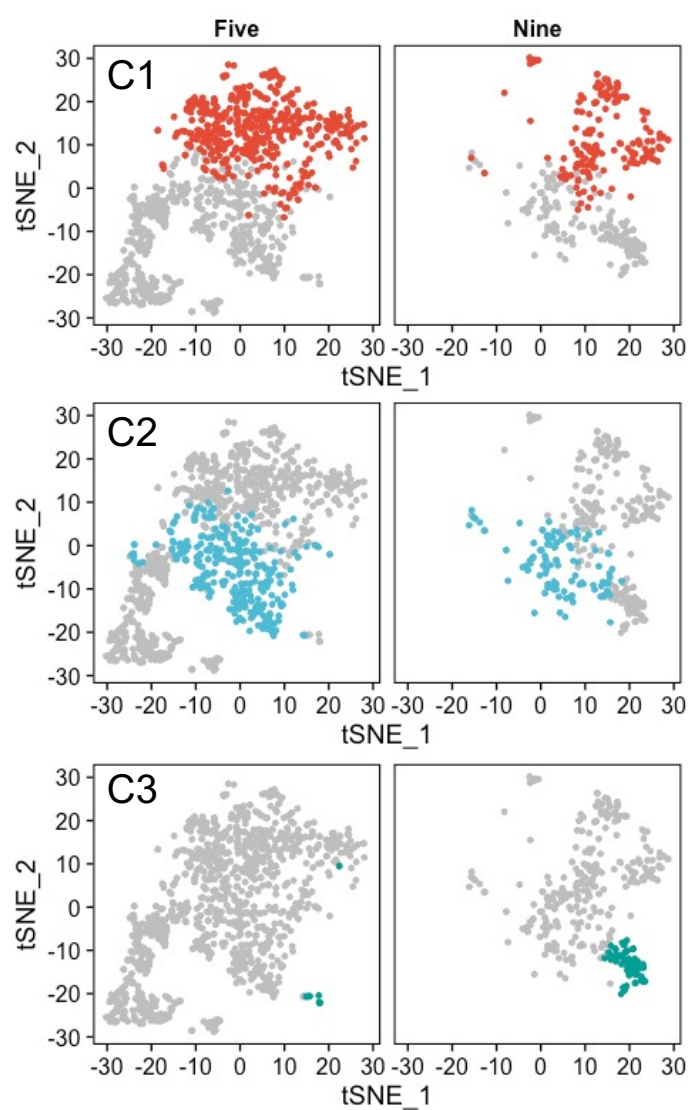

Supplement: Supplementary Figure 6 — Assessment of hypnozoite transcriptomes. (A) UMAP of hypnozoites colored by cluster and faceted by day post-infection. (B) Violin plot showing the average log2FC of differentially expressed genes in each cluster. Markers were identified using Seurat’s AllFindMarkers function. Genes with a Bonferroni adjusted p value < 0.05 (Wilcoxon rank-sum) and average log2FC > 0.5 were deemed markers. Red dashed line: average log2FC = 0.5. (C) Populations identified in the hypnozoite-directed assessment of the data overlaid on the TSNE plot generated for . [file Image_6.pdf]

Figure S7

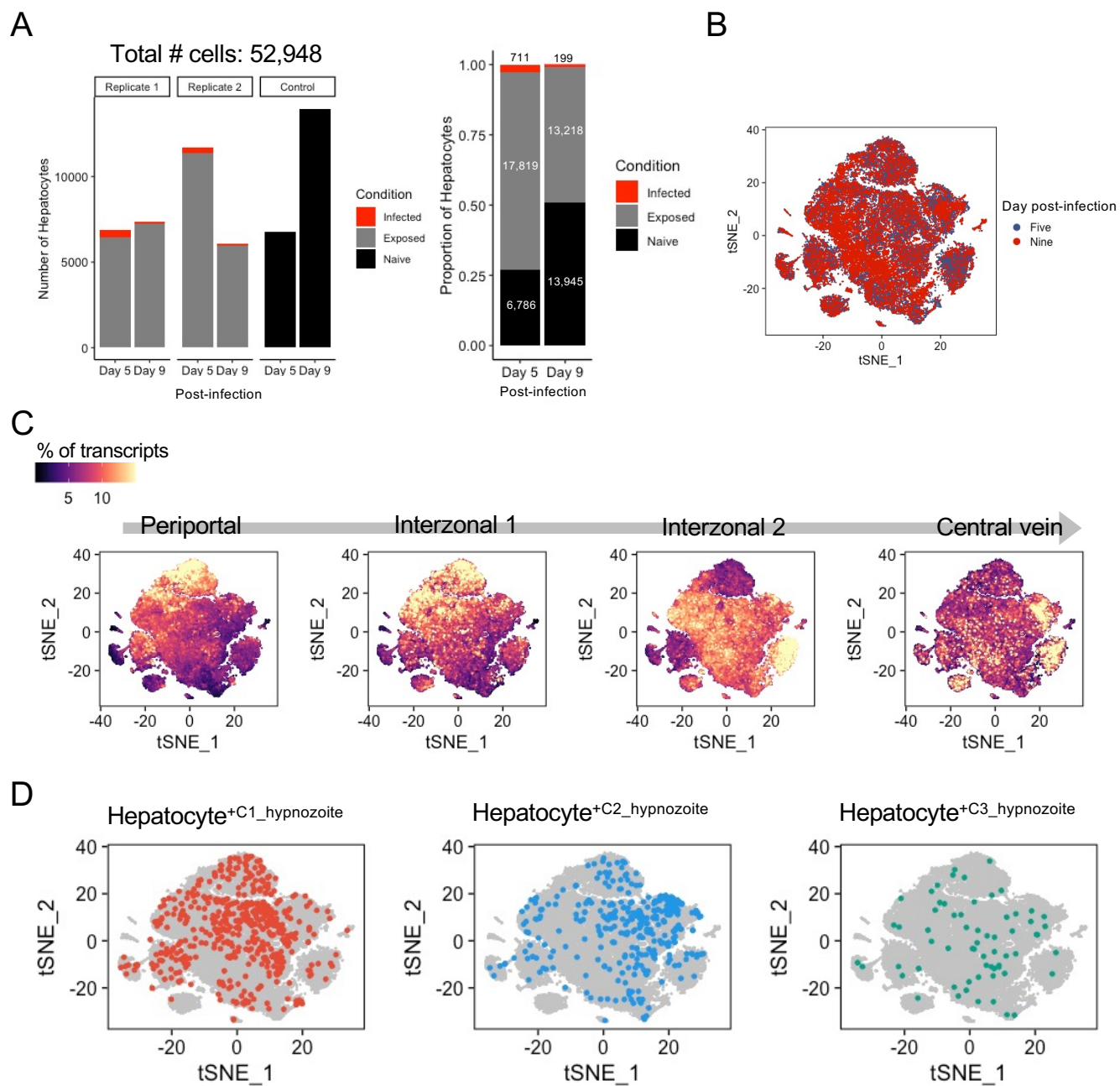

Supplement: Supplementary Figure 7 — Analysis of host-pathogen transcriptional signatures in P. vivax- infected and non-infected hepatocytes. (A) Number of hepatocytes assessed. Colored by infection status and faceted by sample (left); proportion of infected, exposed, or naive hepatocytes assessed on day 5 or day 9 post-infection. (B) t-SNE plot of hepatocytes colored by day. (C) t-SNE plots of hepatocytes colored by percent expression of zonation markers in human hepatocytes. Markers obtained from Macparland et al. (2018). (D) t-SNE plots of hepatocytes colored by hypnozoite infection status. Grey, uninfected (naive and exposed) hepatocytes; red, hepatocytes infected with C1 hypnozoites; blue, hepatocytes infected with C2; teal, hepatocytes infected with C3 hypnozoites. [file Image_7.pdf]

Figure S8

A

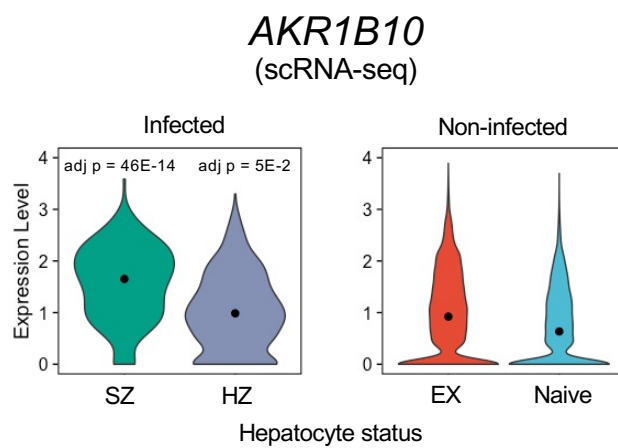

B

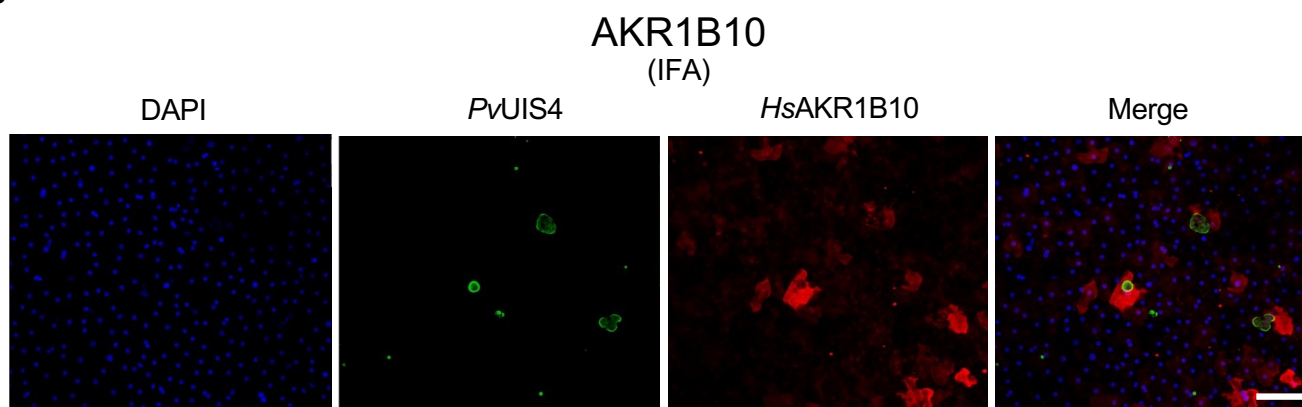

C

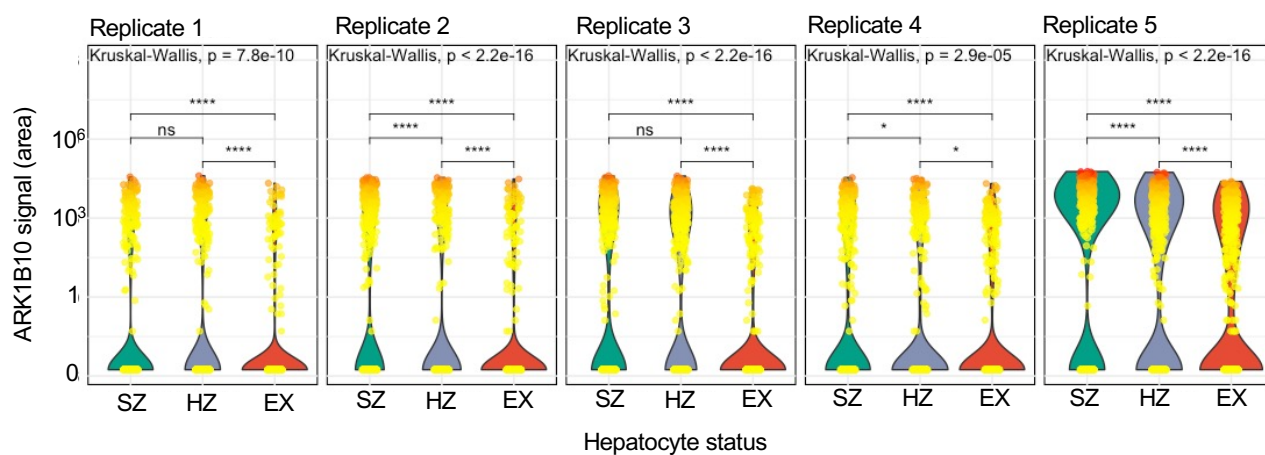

Supplement: Supplementary Figure 8 — Human AKR1B10 is upregulated in hepatocytes infected with P. vivax. (A) Violin plots displaying the distribution of AKR1B10 transcript levels in infected (schizonts and hypnozoites) and non-infected (exposed and naive) hepatocytes. Black dot: mean expression (log2). Adj p: Bonferroni adjusted p value versus non-infected (naïve and exposed) hepatocytes. (B) Representative confocal image from a second biological replicate of P. vivax parasites on day 12 post-infection of hepatocytes. Cells were stained with DAPI (blue), PvUIS4 (green), and HsAKR1B10 (red). White bar represents 100µm. (D) Associated with (right), violins plots displaying the distribution of AKR1B10 signal across all biological replicates. IFA: immunofluorescence assay; SZ: hepatocyte containing schizont; HZ: hepatocyte containing hypnozoite; EX: hepatocyte not infected but exposed to P. vivax. ****: p <= 0.0001; *: p <= 0.05; n.s: not significant, p > 0.05, Wilcoxon test. [file Image_8.pdf]
